# Post-Transplant Cyclophosphamide Allows Allogeneic Hematopoietic Stem-Cell Transplantation Across Donor Types for Nonmalignant Hematologic Diseases

**DOI:** 10.14740/jh2184

**Published:** 2026-04-06

**Authors:** Baldeep Wirk, Xiaoyan Deng

**Affiliations:** a Cellular Immunotherapies and Transplant Program, Massey Comprehensive Cancer Center, Virginia Commonwealth University, Richmond, VA, USA; b Department of Biostatistics, School of Public Health, Virginia Commonwealth University, Richmond, VA, USA

**Keywords:** Aplastic anemia, Diamond-Blackfan anemia, Allogeneic stem-cell transplantation, Conditioning chemotherapy, Graft-versus-host disease

## Abstract

**Background:**

The aim of the study was to compare post-transplant cyclophosphamide (PTCY)-based regimens with historical regimens using calcineurin inhibitor and methotrexate (CNI-MTX) for allogeneic hematopoietic stem-cell transplant (HCT) in nonmalignant hematologic disorders.

**Methods:**

We conducted a single-center, retrospective review of patients with acquired severe aplastic anemia (N = 18) or Diamond-Blackfan anemia (N = 1) who underwent allogeneic HCT from 2011 to 2024. Patients received graft-versus-host disease (GVHD) prophylaxis with either CNI-MTX or PTCY-mycophenolate mofetil-tacrolimus. Primary endpoints were overall survival (OS) and disease-free survival (DFS) without graft failure at 1 year after transplantation.

**Results:**

In the CNI-MTX cohort (N = 14) with severe aplastic anemia, 11 patients received fludarabine-cyclophosphamide-thymoglobulin (ATG)-total body irradiation (TBI), while three received cyclophosphamide-ATG allogeneic HCT. Donors were matched-unrelated (N = 7), matched-related (N = 6), or mismatched-unrelated (N = 1). Graft sources included bone marrow (N = 12) or peripheral blood stem cells (N = 2). One patient developed grade 3 skin acute GVHD, and none had chronic GVHD. There was primary graft failure (N = 6), stable mixed T-cell chimerism (N = 4), and 100% donor chimerism (N = 4). Four patients with primary graft failure underwent salvage second transplants at a median of 103 days (35–322) after the first transplant. Five patients with primary graft failure died at a median of 6 months (0.89–9.3) from the first transplant. The PTCY cohort (N = 5) included four patients with severe aplastic anemia and one with Diamond-Blackfan anemia. All underwent fludarabine-cyclophosphamide-ATG-TBI allogeneic HCT. Donors were matched-related (N = 1), matched-unrelated (N = 2), syngeneic (N = 1), or haploidentical (N = 1). Graft source was peripheral blood stem cells (N = 3) for matched-related, matched-unrelated, and syngeneic transplants, and bone marrow (N = 2) for haploidentical and matched-unrelated donor transplants. Donor chimerism was 100% (N = 3) and mixed chimerism (N = 2). All patients became transfusion-independent, and none developed GVHD or graft failure. The 1-year OS rate was 64.29% vs. 100%, the 1-year DFS rate was 57.14% vs. 100%, and the 1-year GVHD-free, graft failure-free survival (GRFS) was 50% vs.100% for the CNI-MTX and PTCY cohorts, respectively. Despite a trend toward better OS, DFS, and GRFS for PTCY, the OS, DFS, and GRFS time distributions were not statistically significantly different (P = 0.1448, 0.0919, and 0.0627, respectively).

**Conclusion:**

Allogeneic HCT with uniform conditioning of fludarabine-cyclophosphamide-ATG-TBI with PTCY GVHD prophylaxis is effective for adults with severe aplastic anemia or Diamond-Blackfan anemia across donor types (matched-related, syngeneic, matched-unrelated, haploidentical) and should be prospectively compared with historical regimens using CNI-MTX GVHD prophylaxis.

## Introduction

Immunosuppressive therapy for acquired severe aplastic anemia (SAA) with eltrombopag, horse anti-thymocyte globulin, and cyclosporine has a 52% 12-month complete remission rate but a 40% risk of relapse and 15% risk of myeloid neoplasm within 4 years [1–4]. As it can take 3 months to respond to immunosuppressive therapy, the primary cause of death in SAA is infection from prolonged neutropenia [4]. Due to relapse and clonal evolution to myeloid neoplasia with immunosuppressive therapy, the 15-year event-free survival of SAA is only 27% in patients younger than 20 and 12% for those 60 years or older [5]. Thus, immunosuppressive therapy is not curative [5]. Older age exacerbates the risk of relapse and myeloid neoplasia after immunosuppressive therapy [6, 7]. Most patients who progressed to myeloid neoplasia developed myelodysplastic syndrome (75%), followed by acute myeloid leukemia in 18% and myelodysplastic/myeloproliferative neoplasm in 7% [2]. In contrast to *de novo* myelodysplastic syndrome, AA-associated myelodysplastic syndrome had higher revised international prognostic scoring system scores from more frequent *RUNX1* mutations (21% vs. 8%), *ASXL1* mutations (24% vs. 12%), and chromosome 7 abnormalities (53% vs. 11%) [2]. Secondary myeloid neoplasms have poor prognosis even with an allogeneic hematopoietic stem-cell transplantation (HCT). For example, the 2-year leukemia-free survival rate is only 28.5%, and the overall survival (OS) rate is 34.9% with allogeneic HCT for monosomy 7 acute myeloid leukemia [8]. In contrast, upfront allogeneic HCT has curative potential in SAA.

Given the lack of durability with immunosuppressive therapy and the risk of myeloid neoplasia, the American Society for Transplantation and Cellular Therapy recommends upfront allogeneic HCT in fit newly diagnosed acquired SAA patients with matched related donors (MRDs), if available, in those less than 50 years of age, but also in those over 50 years [9]. If an MRD is unavailable, the recommendations are for alternative donor transplants with a matched unrelated donor (MUD) or a haploidentical donor [9]. Upfront MRD-HCT was superior to immunosuppressive therapy with horse ATG, cyclosporine, and eltrombopag in a prospective study of SAA patients [10]. In addition, Xu et al found similar outcomes with upfront haploidentical or MRD HCT. The 9-year OS was 87.1±2.5% for haploidentical donors and 89.3±3.7% for MRD (P = 0.173) [11]. The 9-year disease-free survival (DFS) was 86.5±2.6% for haploidentical donors and 88.1±3.8% for MRD (P = 0.257) [11]. Notably, in a European Society for Blood and Marrow Transplantation meta-analysis of retrospective studies of SAA patients undergoing upfront alternative donor HCT (haploidentical, MUD, mismatched unrelated donor) versus immunosuppressive therapy, the pooled 5-year odds ratio for OS was statistically significant at 0.44 (95% confidence interval (CI), 0.23–0.85) in favor of upfront alternative donor HCT [12].

There are no randomized prospective studies comparing conditioning regimens for transplant in aplastic anemia. In a retrospective registry study from the Center for International Blood and Marrow Transplantation Research (CIBMTR), SAA patients receiving MRD-HCT had similar survival with cyclophosphamide and rabbit antithymocyte globulin (CY-ATG) or fludarabine, cyclophosphamide, and rabbit antithymocyte globulin (FLU-CY-ATG) conditioning [13]. For those undergoing MUD-HCT, the optimal conditioning was FLU-CY-ATG or FLU-CY-ATG with total body irradiation (TBI) [13].

Calcineurin inhibitor and methotrexate (CNI-MTX) remains the standard regimen for graft-versus-host disease (GVHD) prophylaxis in MRD and MUD-HCT. For haploidentical HCT, post-transplant cyclophosphamide (PTCY)-based prophylaxis is preferred. Recently, PTCY-based GVHD prophylaxis was used effectively in MRD and MUD-HCT for SAA patients [14]. There was a 3-year OS of 100% for three SAA patients with MSD, six with MUD, and one with a 7/8 mismatched unrelated donor HCT using the PTCY platform [14]. Furthermore, the low rates of graft rejection and GVHD with PTCY allow for the safe use of alternative donors in transplant for SAA, where the graft-versus-tumor effect is not required [14].

In this study, we compared PTCY-based regimens with historical regimens using CNI-MTX for allogeneic HCT in nonmalignant hematologic disorders to determine whether uniform conditioning and GVHD prophylaxis can be used across donor sources.

## Materials and Methods

### Patients

We reviewed 19 patients with nonmalignant hematologic disorders who underwent allogeneic HCT at the Virginia Commonwealth University between 2011 and 2024. Patients had SAA (N = 18) and Diamond-Blackfan anemia (DBA) (N = 1). The SAA patients had unremarkable testing for inherited bone marrow (BM) failure syndromes. The Virginia Commonwealth University Institutional Review Board (ethics committee) approved this retrospective study. The study adhered to the 1964 Helsinki Declaration and its later amendments.

### Conditioning and GVHD prophylaxis

We compared PTCY-based regimens with historical regimens using CNI-MTX GVHD prophylaxis for allogeneic HCT in nonmalignant hematologic disorders.

The CNI-MTX cohort (N = 14) with SAA had FLU-CY-ATG-TBI (N = 11) or CY-ATG (N = 3) conditioning for allogeneic HCT. The FLU-CY-ATG-TBI regimen consisted of intravenous (IV) fludarabine 30 mg/m^2^ on days −5, −4, −3, −2; cyclophosphamide 50 mg/kg on day −2; rabbit ATG 3 mg/kg on days −4, −3, −2; and TBI 2 Gy on day −1 before transplant. High dose CY-ATG consisted of IV cyclophosphamide 50 mg/kg on day −5 to −2 and rabbit ATG 2.5 mg/kg on day −5 to −3. Standard hydration and MESNA at the equivalent dose to cyclophosphamide were given in both regimens. Unmanipulated grafts of either BM (N = 12) or peripheral blood stem cells (PBSCs) (N = 2) were infused on day 0. From day +4, granulocyte colony-stimulating factor 5 µg/kg/day was given until the absolute neutrophil count (ANC) was > 1 × 10^9^/L for three consecutive days.

Patients received GVHD prophylaxis with a combination of IV tacrolimus 0.015 mg/kg daily beginning on day −3 and methotrexate 15 mg/m^2^ on day 1 and 10 mg/m^2^ on days 3, 6, 11 after transplant with leucovorin rescue. Trough tacrolimus blood levels were maintained at 10 to 15 µg/L for at least a year after transplant to abrogate the risk of late graft failure. Then the tacrolimus was tapered if there was no GVHD.

The PTCY cohort (N = 5) underwent allogeneic HCT with FLU-CY-ATG-TBI consisting of IV rabbit ATG 0.5 mg/kg on day −9 and 2 mg/kg on days −8 and −7; fludarabine 30 mg/m^2^ IV daily from day −6 to day −2; cyclophosphamide 14.5 mg/kg IV daily from day −6 to day −5; and TBI 400 cGy on day −1 before transplant. The unmanipulated donor grafts (three BM and two PBSC) were infused on day 0. On day 5 post-transplant, granulocyte colony-stimulating factor 5 µg/kg/day was given until the ANC was > 1 × 10^9^/L for three consecutive days. Post-transplant GVHD prophylaxis included PTCY 50 mg/kg/day IV on days 3 and 4, mycophenolate mofetil orally 15 mg/kg three times per day up to 1 g three times per day from day 5 to day 35, and tacrolimus orally or IV starting day 5 to maintain a level of 10 to 15 ng/mL for a year and then tapered if there was no GVHD.

All patients received standard antibiotic prophylaxis with acyclovir, levofloxacin, and posaconazole throughout the hospitalization. On day 30 post-transplant, *Pneumocystis jirovecii* prophylaxis with trimethoprim-sulfamethoxazole was begun.

### Endpoints and definitions

All outcomes in the study were defined from the first HCT. The primary endpoints were OS and DFS at 1 year. Events were death from any cause. OS was defined as the time between the HCT and death. Events considered in DFS were primary graft failure. Secondary endpoints included graft failure, grade II-IV acute and chronic GVHD, GVHD-free, relapse-free survival (GRFS), and treatment-related mortality (TRM). The definition of neutrophil engraftment was an ANC > 0.5 × 10^9^/L for three consecutive days. The definition of platelet engraftment was a platelet count > 20 × 10^9^/L without transfusions for the past 7 days. Primary graft failure was an inability to achieve an ANC > 0.5 × 10^9^/L for three consecutive days by day 42 post-transplant. Secondary graft failure was an ANC fall to < 0.5 × 10^9^/L after initial engraftment ANC > 0.5 × 10^9^/L without evidence of any infections or drug toxicity. The definition of GRFS was surviving without primary graft failure, grade 3-4 acute GVHD, or chronic GVHD necessitating systemic therapy. Day 100 post-transplant TRM was defined as death from any cause without graft failure. The definition of acute and chronic GVHD was according to consensus criteria [15–17].

### Chimerism

Pre-transplant specimens obtained from both the recipient and donor established a baseline pattern. A standard silica-based procedure was used to extract the deoxyribonucleic acid (DNA). A polymerase chain reaction (PCR) amplified the DNA using the PowerPlex^®^ 16 HS System (Promega Corporation) to determine informative genetic markers. This information was stored for future comparison with post-transplant specimens from the recipient. Sequential engraftment studies for monitoring used blood or BM specimens. Studies were also performed with specific sorted cell populations such as T-lymphocytes (CD3^+^) and granulocytes (CD66b^+^), using antibody-mediated positive selection and magnetic particle separation. Using fluorescently labeled primers, this system co-amplified the following short tandem repeat (STR) markers (which are repetitive units of 3–7 base pairs): D18S51, D21S11, TH01, D3S1358, Penta E, FGA, TPOX, D8S1179, vWA, Amelogenin, CSF1PO, D16S539, D7S820, D13S317, D5S818, and Penta D. This includes all 13 CODIS STR markers, amelogenin for gender determination, and two penta-nucleotide STR markers. Capillary electrophoresis separated the PCR products. The ChimeRMarker^®^ software (SoftGenetics) analyzed the data. Results were reported as a percentage of donor cells in the post-transplant sample and calculated from the peak area for each informative marker. The linear range of this assay is from 5% to 95% donor cells.

### Statistical analysis

To compare the characteristics of the two cohorts, the *t*-test was used for continuous variables (e.g., age), and Fisher’s exact or the Cochran-Mantel-Haenszel test for categorical variables. To determine whether the OS time distribution differed significantly between the PTCY and CNI-MTX cohorts at 1 year after transplant, an OS analysis was performed using the log-rank test. All 19 patients (PTCY cohort, N = 5; CNI-MTX, N = 14) were included. Any patients who were still alive 1 year after transplant were censored.

Furthermore, to determine whether the DFS time distribution differed significantly between the PTCY and CNI-MTX cohorts at 1 year after transplant, a 1-year DFS analysis was conducted by the log-rank test. All 19 patients (PTCY cohort, N = 5; CNI-MTX cohort, N = 14) were included. Any patients who did not experience graft failure within a year after transplant were censored.

To determine whether the GRFS time distribution differed between the PTCY and CNI-MTX cohorts at a year after the transplant, a 1-year GRFS analysis was conducted using the log-rank test. Any patients who survived without GVHD or graft failure within a year after transplant were censored.

Four Cox proportional hazards models were used to assess the impact of the individual covariates of graft source, age, gender, donor type, conditioning, and chimerism on OS, DFS, and GRFS in the two cohorts.

The analysis was conducted using SAS (Statistical Analysis System) version 9.4 software. The significance level was set to 0.05.

## Results

### CNI-MTX cohort

#### Patient and transplant characteristics

Fourteen patients with acquired SAA underwent allogeneic HCT with CNI-MTX GVHD prophylaxis after FLU-CY-ATG-TBI (N = 11) or CY-ATG (n = 3) conditioning (Table 1). The mean age was 29.1 years (18–58). Thirteen patients had failed initial immunosuppressive therapy with horse ATG and cyclosporine and subsequently had salvage allogeneic HCT. One patient had upfront allogeneic HCT. Median time from diagnosis to transplant for the CNI-MTX cohort was 319 days (range, 37–4,962 days). Donors were MUD (N = 7), MRD (N = 6), and a mismatched unrelated donor (N = 1). Graft source was BM (N = 12) and PBSC (N = 2). Median follow-up was 87.5 months (range, 0.89–181 months).

**Table 1:** Characteristics of Bone Marrow Failure Patients Who Received an Allogeneic Hematopoietic Cell Transplant

Characteristics	PTCY (N = 5)	CNI-MTX (N = 14)	P value
Age, years, mean (minimum–maximum)	33.8 (21–48)	29.1 (18–58)	0.459
Gender, N (%)			0.1409
Female	1 (20)	9 (64.29)	
Male	4 (80)	5 (35.71)	
Diagnosis, N (%)			0.4527
Severe aplastic anemia	4 (80)	14 (100)	
Diamond-Blackfan anemia	1 (20)	0 (0)	
Karnofsky performance status, N (%)			1
90% or more	5 (100)	14 (100)	
Donor type, N (%)			0.1838
Sibling donor	1 (20)	6 (42.86)	
Matched unrelated donor	2 (40)	7 (50)	
Haploidentical	1 (20)	0 (0)	
Identical twin donor (syngeneic)	1 (20)	0 (0)	
Mismatched unrelated donor (7/8)	0 (0)	1 (7.14)	
Graft source, N (%)			0.0844
Bone marrow	2 (40)	12 (85.71)	
Peripheral blood stem cells	3 (60)	2 (14.29)	
Chimerism, N (%)			0.2112
Full donor	2 (40)	4 (28.57)	
Mixed T-cell chimerism; full donor myeloid chimerism	2 (40)	4 (28.57)	
None (primary graft failure)	0 (0)	6 (42.86)	
Syngeneic donor	1 (20)	0	
Conditioning, N (%)			0.2723
Cyclophosphamide and rabbit ATG	0 (0)	3 (21.43)	
Fludarabine, cyclophosphamide, rabbit ATG, TBI	5 (100)	11 (78.57)	

ATG: rabbit anti-thymocyte globulin; CNI: calcineurin inhibitor; MTX: methotrexate; PTCY: post-transplant cyclophosphamide; TBI: total body irradiation.

#### Outcomes with CNI-MTX GVHD prophylaxis

Six patients (42%) had primary graft failure. An additional patient had secondary graft failure necessitating salvage allogeneic HCT four and a half years from the first transplant. There was primary graft failure (N = 6), mixed chimerism (N = 4), and 100% donor chimerism (N = 4). Four patients with primary graft failure had salvage second allogeneic HCT at a median of 103 days (35–322) after the first transplant. Five patients died at a median 6 months (range, 0.89–9.3) from the first transplant due to primary graft failure. One patient had grade 3 skin acute GVHD at day 72 post-transplant. None had chronic GVHD. Overall, the day 100 TRM was 0. Eight patients are alive at a median follow-up of 120.5 months (range, 48–181 months) and are transfusion-independent. BM next-generation sequencing (NGS) before and after transplant was not available.

### PTCY cohort

#### Patient and transplant characteristics

The PTCY cohort (N = 5) underwent FLU-CY-ATG-TBI 4 Gy allogeneic HCT for SAA (N = 4) and DBA (N = 1). Mean age was 33.8 years (21–48) (Table 2). Three SAA patients had upfront transplant at a median of 118 days (range, 105–143) from diagnosis. The DBA patient had an MUD-HCT at age 35 after failing to respond to prednisone. Another SAA patient relapsed 2 years after immunosuppressive therapy (horse ATG, cyclosporine, and eltrombopag) and had haploidentical BM transplantation 814 days from diagnosis. Median time from diagnosis to transplant for the PTCY cohort was 143 days (range, 105–12,775 days). Donors were MRD (N = 1), MUD (N = 2), syngeneic (N = 1), and haploidentical (N = 1). Graft sources were PBSC (N = 3) in MRD, MUD, and syngeneic allogeneic HCT, and BM (N = 2) in haploidentical and MUD allogeneic HCT. Median follow-up was 17.6 months (range, 15.6–20.7 months).

**Table 2:** Characteristics of Bone Marrow Failure Patients Who Received Allogeneic Hematopoietic Cell Transplantation With Post-Transplant Cyclophosphamide-Based Conditioning

Age/gender/diagnosis	Prior therapy	Diagnosis to transplant (days)	CD34^+^/kg infused	Donor/graft source	Neutrophil engraftment (days)	Platelet engraftment (days)	Acute GVHD	Chronic GVHD	Chimerism at day 100 Myeloid/T-cell	Infection by day 100	F/U, months
22 years Male SAA	None	105	5.96	Syngeneic PBSC	14	18	No	No	N/A	No	13.6
35 years Male DBA	Prednisone	12,775	5.94	MUD PBSC	14	14	No	No	100%/100%	No	15
48 years Male SAA	None	118	5.96	MRD PBSC	17	31	No	No	100%/93%	No	15.4
43 Male SAA	None	143	2.16	MUD BM	13	18	No	No	100%/81%	No	17.8
21 years Female SAA	ATG, CSA, Eltrombopag	814	2.59	Haploidentical related BM	12	18	No	No	100%/100%	No	18.7

ATG: horse anti-thymocyte globulin; BM: bone marrow; CSA: cyclosporine; DBA: Diamond-Blackfan anemia; GVHD: graft-versus-host disease; MRD: matched related donor; MUD: matched unrelated donor; PBSCs: peripheral blood stem cells; SAA: severe aplastic anemia; F/U: follow-up.

#### Outcomes with PTCY GVHD prophylaxis

Two patients had 100% donor chimerism, and two had stable mixed T-cell chimerism with complete myeloid chimerism and normal blood cell counts. Chimerism studies could not be performed in the patient with a syngeneic donor. The syngeneic donor was identified based on the history of a monochorionic placenta. The patient achieved normal blood counts. All patients became transfusion-independent. None had acute or chronic GVHD or graft failure. Neutrophil engraftment occurred at a median of 14 days (range, 12–17). Platelet engraftment occurred at a median of 18 days (range, 14–31). The three SAA patients who had upfront allogeneic HCT had negative BM NGS before and after transplantation. The SAA patient who received initial immunosuppressive therapy had pre-transplant NGS showing an IKZF1 mutation, whereas the post-transplant NGS was negative. The DBA patient had a *SH2B3* mutation on pre-transplant NGS, but post-transplant NGS was negative. The day 100 TRM was 0. No infections occurred by 1-year follow-up. All patients are alive at a median follow-up of 17.6 months (range, 15.6–20.7 months).

### Comparing the CN-MTX and PTCY cohorts

**Figure 1:**
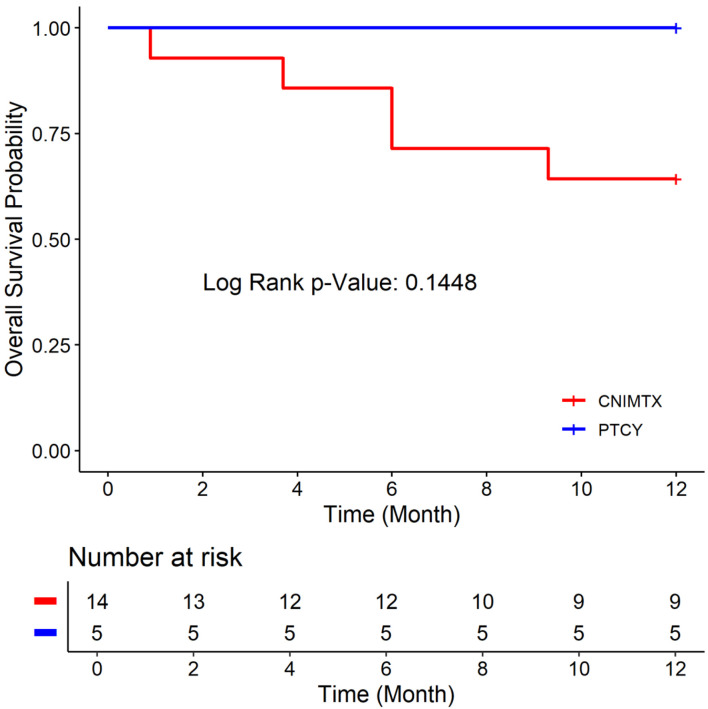
Overall survival of the two cohorts: post-transplant cyclophosphamide (PTCY) versus calcineurin inhibitor and methotrexate (CNI-MTX).

**Figure 2:**
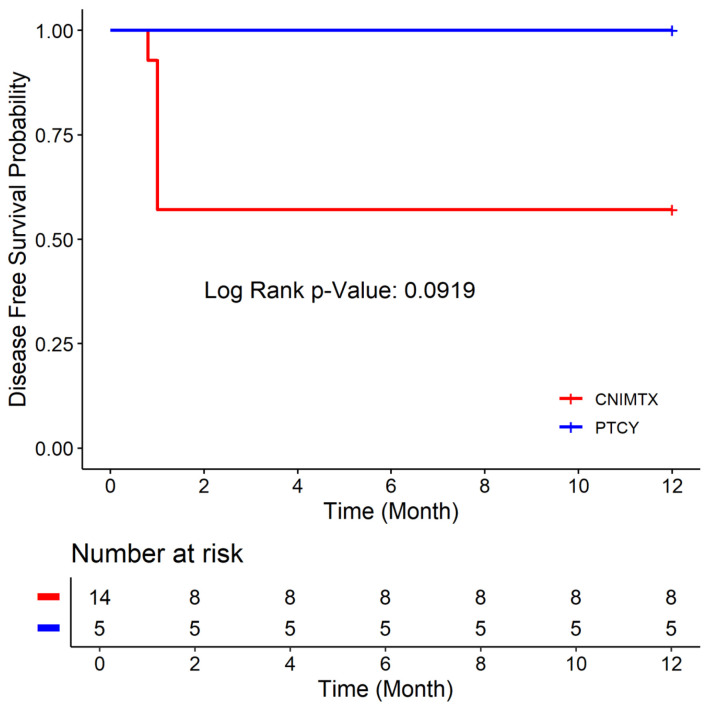
Disease-free survival of the two cohorts: post-transplant cyclophosphamide (PTCY) versus calcineurin inhibitor and methotrexate (CNI-MTX).

**Figure 3:**
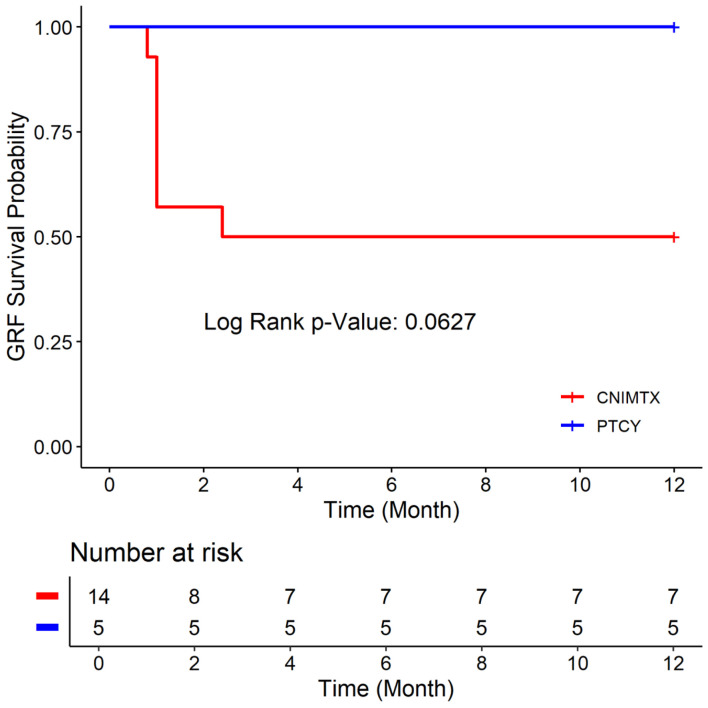
Graft-versus-host disease-free, relapse-free survival (GRFS) of the two cohorts: post-transplant cyclophosphamide (PTCY) versus calcineurin inhibitor and methotrexate (CNI-MTX).

There was no statistically significant difference in the age, Karnofsky performance status, donor type, conditioning chemotherapy, or graft source between the CNI-MTX and PTCY cohorts (Table 1). The 1-year OS rate was 64.29% vs. 100% (Fig. 1), the 1-year DFS rate was 57.14% vs. 100% (Fig. 2), and the 1-year GRFS was 50% vs.100% (Fig. 3) for the CNI-MTX and PTCY cohorts, respectively. Despite a trend toward better OS, DFS, and GRFS for PTCY, the OS, DFS, and GRFS time distributions between the two cohorts were not statistically significantly different with log-rank test P values of 0.1448, 0.0919, and 0.0627, respectively (Supplementary Materials 1–9, jh.elmerpub.com). The cumulative incidence of graft failure did not significantly differ between the CNI-MTX and PTCY cohorts (Gray’s test P value 0.059). There was no significant impact of the covariates of graft source, age, gender, chimerism, donor type, or conditioning on the OS, DFS, or GRFS time distributions in the two cohorts (Supplementary Materials 3, 6, and 9, jh.elmerpub.com).

## Discussion

We compared PTCY-based regimens to historical regimens using CNI-MTX for allogeneic HCT in nonmalignant hematologic disorders. The age, Karnofsky performance status, donor type, or graft source (PBSC or BM) did not differ between the CNI-MTX and PTCY cohorts. The 1-year OS rate was 64.29% vs. 100%, the 1-year DFS rate was 57.14% vs. 100%, and the 1-year GRFS was 50% vs. 100% for the CNI-MTX and PTCY cohorts, respectively. Despite a trend toward better OS, DFS, and GRFS for PTCY, the OS, DFS, and GRFS time distributions between the two cohorts were not statistically significantly different. Even so, five patients died at a median of 6 months (0.89–9.3) from the first transplant due to primary graft failure in the CNI-MTX cohort. None died in the PTCY cohort at the time of this report. Four patients in the CNI-MTX cohort and two in the PTCY cohort had split chimerism with complete myeloid chimerism but mixed T-cell chimerism, as often seen with ATG-based conditioning regimens. The patients maintained normal blood counts and did not require intervention.

There are no randomized prospective studies directly comparing conditioning regimens for transplant in aplastic anemia, given the disease’s rarity. Nor are there studies directly comparing GVHD prophylaxis with CNI-MTX versus PTCY in aplastic anemia. The British Society for Haematology recommends CNI-MTX for MRD and MUD HCT and PTCY-based GVHD prophylaxis for haploidentical HCT in SAA [18]. Whereas the American Society for Transplantation and Cellular Therapy recommends CNI-MTX or PTCY-based GVHD prophylaxis for SAA patients undergoing MRD or MUD-HCT and PTCY-based prophylaxis for those receiving haploidentical HCT [9]. Our data further support these recommendations by showing PTCY-based prophylaxis is effective in haploidentical, MRD, and MUD-HCT for aplastic anemia. Our study found no difference in OS, DFS, or GRFS between CNI-MTX and PTCY-based GVHD prophylaxis.

In our study, uniform PTCY-based conditioning with FLU-CY-ATG-TBI 4Gy in MRD (N = 1), MUD (N = 2), syngeneic (N = 1), and haploidentical (N = 1) allogeneic HCT offered 100% 1-year OS, DFS, and GRFS. No patient had graft failure. The patient with DBA underwent MUD-HCT at age 35, by which time he had transfusional iron overload in the liver and heart. Comorbidities such as these in adults preclude myeloablative conditioning, the current standard for DBA [19]. With the reduced-intensity regimen of FLU-CY-ATG-TBI 4 Gy with PTCY, mycophenolate mofetil, and tacrolimus, he achieved complete donor chimerism without GVHD and is transfusion-independent for more than a year after transplantation.

Without conditioning, the graft failure rate in syngeneic transplants for aplastic anemia is 64% [20]. The risk of graft failure with syngeneic donors also increases with the use of BM stem cells and the lack of post-transplant immunosuppression [20]. Data on the optimal conditioning for syngeneic HCT in aplastic anemia are limited. In our study, uniform PTCY-based conditioning with FLU-CY-ATG-TBI 4 Gy enabled sustained engraftment and no graft failure in the SAA patient who received a syngeneic peripheral blood stem-cell transplant.

Given the rarity of aplastic anemia, there are no randomized prospective studies comparing conditioning regimens for transplant. Our study is limited by its retrospective nature and small sample size. Even though the OS, DFS, and GRFS showed a trend in favor of the PTCY cohort, there was no statistically significant difference in these outcomes at 12 months after transplant for the PTCY and CNI-MTX cohorts, perhaps due to the limited sample size. Confounding variables, including graft source, age, conditioning regimens, and donor type, were analyzed. There was no significant impact of the covariates of graft source, age, gender, conditioning regimens, donor type, or chimerism on the OS, DFS, and GRFS time distributions in the two cohorts. Despite these limitations, transplant outcomes with uniform conditioning and PTCY-based GVHD prophylaxis across various donor sources (MRD, MUD, syngeneic, haploidentical) were encouraging and comparable to CNI-MTX GVHD prophylaxis in aplastic anemia.

The Blood and Marrow Transplant Clinical Trials Network (BMT CTN) 1203 study compared three GVHD prophylaxis regimens in patients with hematologic malignancies undergoing MRD, 8/8 MUD, or 7/8 MUD allogeneic peripheral blood stem-cell transplantation [21]. The GVHD regimens were PTCY-tacrolimus-mycophenolate mofetil, bortezomib-tacrolimus-methotrexate, and maraviroc-tacrolimus-methotrexate. Each GVHD prophylaxis regimen was compared with a nonrandomized control group receiving standard tacrolimus-methotrexate GVHD prophylaxis. The PTCY cohort demonstrated superior GRFS and was the only regimen to outperform the nonrandomized tacrolimus-methotrexate control group [21]. BMT CTN 1703 confirmed these results in a phase III, randomized, prospective study in patients with hematologic malignancies receiving MRD, 8/8 MUD, or 7/8 MUD allogeneic peripheral blood stem-cell transplantation [22]. This study compared PTCY-tacrolimus-mycophenolate mofetil to tacrolimus-methotrexate GVHD prophylaxis [22]. The 2-year GRFS with PTCY-tacrolimus-mycophenolate mofetil was significantly better (42.4% vs. 28.8%, P = 0.001). However, there was no difference in OS, DFS, or TRM between the two cohorts [22].

In non-malignant hematologic disorders, we await the results from the CUREAA prospective study of PTCY-based GVHD prophylaxis. These results could confirm the promising findings from retrospective studies and may be practice-changing [23, 24]. CUREAA is a phase II single-arm study in adults older than 25 years with newly diagnosed SAA without matched related donors. The primary endpoint of the study is GRFS of upfront haploidentical, 8/8 MUD, or 7/8 MUD BM transplant using FLU-CY-ATG-TBI 4 Gy uniform conditioning with PTCY-tacrolimus-mycophenolate mofetil GVHD prophylaxis.

In conclusion, allogeneic stem-cell transplantation with uniform conditioning of fludarabine-cyclophosphamide-ATG-TBI 4 Gy and PTCY GVHD prophylaxis is effective for adults with SAA or DBA across multiple donor types (matched-related, syngeneic, matched-unrelated, haploidentical) and should be compared with historical regimens using CNI-MTX GVHD prophylaxis.

## Supplementary Material

Suppl 1 Overall survival at 12 months by cohort (PTCY versus CNI-MTX).

Suppl 2 Test of overall survival time distribution equality over cohorts.

Suppl 3 Six Cox models used to analyze the impact of the individual covariates of graft source, gender, age, donor type, conditioning, and chimerism on the overall survival time distribution for the two cohorts (PTCY versus CNI-MTX).

Suppl 4 Disease-free survival at 12 months by cohort (PTCY versus CNI-MTX).

Suppl 5 Test of disease-free survival time distribution equality over cohorts.

Suppl 6 Six Cox models used to analyze the impact of the individual covariates of graft source, gender, age, donor type, conditioning, and chimerism on the disease-free survival time distribution for the two cohorts (PTCY versus CNI-MTX).

Suppl 7 Graft-versus-host disease-free, relapse-free survival (GRFS) at 12 months by cohort (PTCY versus CNI-MTX).

Suppl 8 Test of GRFS time distribution equality over cohorts.

Suppl 9 Six Cox models used to analyze the impact of the individual covariates of graft source, gender, age, donor type, conditioning, and chimerism on the GRFS time distribution for the two cohorts (PTCY versus CNI-MTX).

## Data Availability

The authors declare that data supporting the findings of this study are available within the article.

## References

[1] Gurnari C, Pagliuca S, Maciejewski JP (2023). Clonal evolution in aplastic anemia: failed tumor surveillance or maladaptive recovery?. Leuk Lymphoma.

[2] Gurnari C, Pagliuca S, Prata PH, Galimard JE, Catto LFB, Larcher L, Sebert M (2023). Clinical and molecular determinants of clonal evolution in aplastic anemia and paroxysmal nocturnal hemoglobinuria. J Clin Oncol.

[3] Peffault de Latour R, Kulasekararaj A, Iacobelli S, Terwel SR, Cook R, Griffin M, Halkes CJM (2022). Eltrombopag added to immunosuppression in severe aplastic anemia. N Engl J Med.

[4] Patel BA, Groarke EM, Lotter J, Shalhoub R, Gutierrez-Rodrigues F, Rios O, Quinones Raffo D (2022). Long-term outcomes in patients with severe aplastic anemia treated with immunosuppression and eltrombopag: a phase 2 study. Blood.

[5] Tichelli A, de Latour RP, Passweg J, Knol-Bout C, Socie G, Marsh J, Schrezenmeier H (2020). Long-term outcome of a randomized controlled study in patients with newly diagnosed severe aplastic anemia treated with antithymocyte globulin and cyclosporine, with or without granulocyte colony-stimulating factor: a Severe Aplastic Anemia Working Party Trial from the European Group of Blood and Marrow Transplantation. Haematologica.

[6] Townsley DM, Scheinberg P, Winkler T, Desmond R, Dumitriu B, Rios O, Weinstein B (2017). Eltrombopag added to standard immunosuppression for aplastic anemia. N Engl J Med.

[7] Olnes MJ, Scheinberg P, Calvo KR, Desmond R, Tang Y, Dumitriu B, Parikh AR (2012). Eltrombopag and improved hematopoiesis in refractory aplastic anemia. N Engl J Med.

[8] Poire X, Labopin M, Polge E, Volin L, Finke J, Ganser A, Blaise D (2020). The impact of concomitant cytogenetic abnormalities on acute myeloid leukemia with monosomy 7 or deletion 7q after HLA-matched allogeneic stem cell transplantation. Am J Hematol.

[9] Iftikhar R, DeFilipp Z, DeZern AE, Pulsipher MA, Bejanyan N, Burroughs LM, Kharfan-Dabaja MA (2024). Allogeneic hematopoietic cell transplantation for the treatment of severe aplastic anemia: evidence-based guidelines from the american society for transplantation and cellular therapy. Transplant Cell Ther.

[10] Liu L, Lei M, Fu R, Han B, Zhao X, Liu R, Zhang Y (2022). Matched related transplantation versus immunosuppressive therapy plus eltrombopag for first-line treatment of severe aplastic anemia: a multicenter, prospective study. J Hematol Oncol.

[11] Xu ZL, Xu LP, Wu DP, Wang SQ, Zhang X, Xi R, Gao SJ (2022). Comparable long-term outcomes between upfront haploidentical and identical sibling donor transplant in aplastic anemia: a national registry-based study. Haematologica.

[12] Alotaibi H, Aljurf M, de Latour R, Alfayez M, Bacigalupo A, Fakih RE, Schrezenmeier H (2022). Upfront alternative donor transplant versus immunosuppressive therapy in patients with severe aplastic anemia who lack a fully HLA-matched related donor: systematic review and meta-analysis of retrospective studies, on behalf of the severe aplastic anemia working party of the European group for blood and marrow transplantation. Transplant Cell Ther.

[13] Bejanyan N, Kim S, Hebert KM, Kekre N, Abdel-Azim H, Ahmed I, Aljurf M (2019). Choice of conditioning regimens for bone marrow transplantation in severe aplastic anemia. Blood Adv.

[14] DeZern AE, Zahurak M, Symons HJ, Cooke KR, Huff CA, Jain T, Swinnen LJ (2023). Alternative donor BMT with posttransplant cyclophosphamide as initial therapy for acquired severe aplastic anemia. Blood.

[15] Przepiorka D, Weisdorf D, Martin P, Klingemann HG, Beatty P, Hows J, Thomas ED (1995). 1994 consensus conference on acute GVHD grading. Bone Marrow Transplant.

[16] Shulman HM, Sullivan KM, Weiden PL, McDonald GB, Striker GE, Sale GE, Hackman R (1980). Chronic graft-versus-host syndrome in man. A long-term clinicopathologic study of 20 Seattle patients. Am J Med.

[17] MacMillan ML, Weisdorf DJ, Wagner JE, DeFor TE, Burns LJ, Ramsay NK, Davies SM (2002). Response of 443 patients to steroids as primary therapy for acute graft-versus-host disease: comparison of grading systems. Biol Blood Marrow Transplant.

[18] Kulasekararaj A, Cavenagh J, Dokal I, Foukaneli T, Gandhi S, Garg M, Griffin M (2024). Guidelines for the diagnosis and management of adult aplastic anaemia: A British Society for Haematology Guideline. Br J Haematol.

[19] Diaz-de-Heredia C, Bresters D, Faulkner L, Yesilipek A, Strahm B, Miano M, Dalle JH (2021). Recommendations on hematopoietic stem cell transplantation for patients with Diamond-Blackfan anemia. On behalf of the Pediatric Diseases and Severe Aplastic Anemia Working Parties of the EBMT. Bone Marrow Transplant.

[20] Gerull S, Stern M, Apperley J, Beelen D, Brinch L, Bunjes D, Butler A (2013). Syngeneic transplantation in aplastic anemia: pre-transplant conditioning and peripheral blood are associated with improved engraftment: an observational study on behalf of the Severe Aplastic Anemia and Pediatric Diseases Working Parties of the European Group for Blood and Marrow Transplantation. Haematologica.

[21] Bolanos-Meade J, Reshef R, Fraser R, Fei M, Abhyankar S, Al-Kadhimi Z, Alousi AM (2019). Three prophylaxis regimens (tacrolimus, mycophenolate mofetil, and cyclophosphamide; tacrolimus, methotrexate, and bortezomib; or tacrolimus, methotrexate, and maraviroc) versus tacrolimus and methotrexate for prevention of graft-versus-host disease with haemopoietic cell transplantation with reduced-intensity conditioning: a randomised phase 2 trial with a non-randomised contemporaneous control group (BMT CTN 1203). Lancet Haematol.

[22] Holtan SG, Bolanos-Meade J, Al Malki MM, Wu J, Kitko CL, Reshef R, Rezvani AR (2025). Improved patient-reported outcomes with post-transplant cyclophosphamide: a quality-of-life evaluation and 2-year outcomes of BMT CTN 1703. J Clin Oncol.

[23] Wirk B (2024). Acquired aplastic anemia therapies: immunosuppressive therapy versus alternative donor hematopoietic cell transplantation. J Hematol.

[24] Muffly L (2023). Two national clinical trials poised to expand the role of bone marrow transplant in newly diagnosed severe aplastic anemia. The Hematologist.

