# Supplementary material for: Post-Transplant Cyclophosphamide Allows Allogeneic Hematopoietic Stem-Cell Transplantation Across Donor Types for Nonmalignant Hematologic Diseases
Source: J Hematol. 2026 Apr 6;15(2):71–9. doi: 10.14740/jh2184 (PMC13071939; doi:10.14740/jh2184)
Supplement: Suppl 2 — Test of overall survival time distribution equality over cohorts. [file jh-15-02-071-s002.docx]

Suppl 2. Test of overall survival time distribution equality over cohorts.

| Test | Chi-square | Degree of Freedom | Probability> Chi-square |
| --- | --- | --- | --- |
| Log-Rank | 2.1261 | 1 | 0.1448 |
| Wilcoxon | 2.1008 | 1 | 0.1472 |
| -2Log(LR)* | 3.7027 | 1 | 0.0543 |
