# Supplementary material for: Post-Transplant Cyclophosphamide Allows Allogeneic Hematopoietic Stem-Cell Transplantation Across Donor Types for Nonmalignant Hematologic Diseases
Source: J Hematol. 2026 Apr 6;15(2):71–9. doi: 10.14740/jh2184 (PMC13071939; doi:10.14740/jh2184)
Supplement: Suppl 4 — Disease-free survival at 12 months by cohort (PTCY versus CNI-MTX). [file jh-15-02-071-s004.docx]

Suppl 4. Disease-Free Survival at 12 months by cohort (PTCY versus CNI-MTX)

| STRATUM | COHORT | TOTAL | FAILED | CENSORED N (%) |
| --- | --- | --- | --- | --- |
| 1 | PTCY | 5 | 0 | 5 (100%) |
| 2 | CNI-MTX | 14 | 6 | 8 (57.14%) |
| Total |  | 19 | 6 | 13 (68.42%) |
