# Supplementary material for: Post-Transplant Cyclophosphamide Allows Allogeneic Hematopoietic Stem-Cell Transplantation Across Donor Types for Nonmalignant Hematologic Diseases
Source: J Hematol. 2026 Apr 6;15(2):71–9. doi: 10.14740/jh2184 (PMC13071939; doi:10.14740/jh2184)
Supplement: Suppl 6 — Six Cox models used to analyze the impact of the individual covariates of graft source, gender, age, donor type, conditioning, and chimerism on the disease-free survival time distribution for the two cohorts (PTCY versus CNI-MTX). [file jh-15-02-071-s006.docx]

Suppl 6. Six Cox models used to analyze the impact of the individual covariates of graft source, gender, age, donor type, conditioning, and chimerism on the disease-free survival time distribution for the two cohorts (PTCY versus CNI-MTX).

| **EFFECT** | **Degree of Freedom** | **Wald Chi-Square** | **Probability> Chi-square** |
| --- | --- | --- | --- |
| *COHORT* | 1 | 0 | 0.9948 |
| Graft source | 1 | 0.01 | 0.9204 |
| *COHORT* | 1 | 0 | 0.9949 |
| Gender | 1 | 0.0554 | 0.814 |
| *COHORT* | 1 | 0 | 0.9949 |
| Age years | 1 | 0.903 | 0.342 |
| *COHORT*  Donor type | 1  4 | 0  0.8049 | 0.9964  0.9378 |
| *COHORT*  Conditioning | 1  1 | 0  0.1267 | 0.9949  0.7218 |
| *COHORT* | 1 | 0 | 1 |
| Chimerism | 2 | 0 | 1 |
