# Supplementary material for: Post-Transplant Cyclophosphamide Allows Allogeneic Hematopoietic Stem-Cell Transplantation Across Donor Types for Nonmalignant Hematologic Diseases
Source: J Hematol. 2026 Apr 6;15(2):71–9. doi: 10.14740/jh2184 (PMC13071939; doi:10.14740/jh2184)
Supplement: Suppl 9 — Six Cox models used to analyze the impact of the individual covariates of graft source, gender, age, donor type, conditioning, and chimerism on the GRFS time distribution for the two cohorts (PTCY versus CNI-MTX). [file jh-15-02-071-s009.docx]

Suppl 9. Six Cox models used to analyze the impact of the individual covariates of graft source, gender, age, donor type, conditioning, and chimerism on the GRFS time distribution for the two cohorts (PTCY versus CNI-MTX).

| **EFFECT** | **Degree of Freedom** | **Wald Chi-Square** | **Probability> Chi-square** |
| --- | --- | --- | --- |
| *COHORT* | 1 | 0 | 0.9962 |
| Graft source | 1 | 0.9917 | 0.3193 |
| *COHORT* | 1 | 0 | 0.9964 |
| Gender | 1 | 0.2567 | 0.6124 |
| *COHORT* | 1 | 0 | 0.9963 |
| Age years | 1 | 0.581 | 0.4459 |
| *COHORT*  Donor type | 1  4 | 0  0.6584 | 0.9959  0.9564 |
| *COHORT*  Conditioning | 1  1 | 0  0.2985 | 0.9964  0.5848 |
| *COHORT* | 1 | 0 | 0.9981 |
| Chimerism | 2 | 0 | 1 |
